# Made in Europe: will artemisinin resistance emerge in French Guiana?

**DOI:** 10.1186/1475-2875-12-152

**Published:** 2013-05-04

**Authors:** Mathieu Nacher, Philippe J Guérin, Magalie Demar-Pierre, Félix Djossou, François Nosten, Bernard Carme

**Affiliations:** 1 Centre d’Investigation Clinique Epidémiologie Clinique Antilles Guyane CIC-EC CIE 802, Cayenne General Hospital, Cayenne, French Guiana; 2 Equipe EPaT EA3593 Epidémiologie des Parasitoses et Mycoses Tropicales, Universite Antilles Guyane, Martinique, Antilles; 3 World Wide Antimalarial Resistance Network (WWARN) and Nuffield Department of Clinical Medicine, University of Oxford, Old Road, Oxford, OX3 7LJ, UK; 4 Unité des Maladies Infectieuses et Tropicales, Centre Hospitalier de Cayenne, French Guiana, France; 5 Shoklo Malaria Research Unit, Mahidol-Oxford Tropical Medicine Research Unit, Bangkok, Thailand; 6 Laboratoire Hospitalo Universitaire de Parasitologie Mycologie, Centre Hospitalier de Cayenne, French Guiana, France

## Abstract

Resistance to artemisinin casts a shadow on the fight against malaria. The importance of illegal gold miners and of malaria in isolated regions of French Guiana constitutes a threat that endangers the fight against malaria in the Amazon. The hurdles of French laws and the remoteness of the territory from France make it impossible for the system to adapt to the problem of total inaccessibility of an important part of the malaria problem. Transmission is high in these areas and gold miners self-medicate with erratic regimens of artemisinin combinations, thus creating perfect conditions for the emergence of resistance. What needs to be done is being done, but within the limits of national law, with some results. However, facing the same difficult problem, Suriname shows more flexibility and is doing much better than French Guiana despite having lower resources. Local authorities in French Guiana cannot overrule the laws that block appropriate malaria care from reaching a third of malaria-exposed persons. Thus the health authorities in France should take immediate calibrated legislative and financial measures to avoid a predictable disaster.

## Background

Encouraged by the last decade’s funding efforts and significant successes, malaria elimination has become a serious goal. In the past, such efforts have stumbled on the rise of resistance to anti-malarials and insecticides that affected the sustainability of the progresses made. Now, the recent successes of artemisinin-based combinations are increasingly threatened by the spread of artemisinin resistance [1,2], thus casting a shadow on recent optimism.

In the late 1950s, shortly after WHO embarked on the malaria eradication programme, resistance of *Plasmodium falciparum* to chloroquine emerged on the Thai-Cambodia border and spread throughout Asia and then Africa within decades. Resistance to chloroquine also appeared independently in the Amazon Basin. The same pattern happened again for the next anti-malarial drug, sulphadoxine+pyrimethamine. The emergence was in Southeast Asia and it spread towards Africa. Again, resistance independently appeared in the Amazon Basin and spread to the subcontinent. Now the hopes of controlling and eliminating malaria are threatened by resistance against artemisinin derivatives, which has emerged and is spreading in Southeast Asia [1-3]. There are reasons to fear that it will also emerge in the Amazon Basin which holds 98% of *P. falciparum* malaria cases in South America, and notably the Guiana shield where the majority of cases are concentrated [4].

### Looking for gold, finding malaria

Throughout the Amazon Basin, there has been remarkable progress in controlling malaria. However, the problem of mobile populations, such as miners, poses a challenge in the region. An additional challenge is that these mobile populations cross borders, and thus interventions of the different countries should be coherent and coordinated in order to treat the problem globally. The common features of Southeast Asia and the Amazon are that malaria transmission often affects remote forested areas where the reach of health care systems is challenged. In addition, the people affected are often mobile and engaged in illegal activities, such as gold or gem mining, logging or fighting. In these areas there is no vector control, and ill persons often self medicate with erratic drug regimens, often using single drugs of variable quality, although for this the situation in South America seems to be better than in Southeast Asia [5,6].

There are reasons to believe that resistance to artemisinin will appear in Europe’s own back yard. Following the recent global economic meltdown, the price of gold has sky rocketed, thus fuelling another gold rush. There are gold miners, and there is malaria in the Amazon and the Guianas. An estimated 10,000-15,000 *garimpeiros* mostly originating from northern Brazil illegally operate in French Guiana with profound consequences on human health, the environment, security and the economy [7]. The *garimpeiros’s* health-seeking behaviour in French Guiana is not uniform. First, they are often far away from health structures. Both Suriname [4] and Brazil report that a significant number of people cross the border to get diagnosed and treated for malaria, allegedly to avoid being arrested in French villages by the authorities. When they get malaria, part of the treatment they receive is taken and part is reportedly sold back on the mining sites in exchange for gold. Remote health centres in French Guiana also report treating *garimpeiros* with falciparum malaria using Riamet® (fixed combination of artemether-lumefantrine, Novartis). Finally, gold miners also have access to artemisinin-based combination of unknown origin (Artecom® is neither commercialized in Suriname, in French Guiana nor in Brazil) on the gold mining sites, as was observed during “Harpie” operations by the French military.

### Neighbouring successes and French legal constraints

Fighting malaria in mobile populations, hidden in the tropical forest, is a very difficult challenge, for all health delivery systems. The problem is compounded by the fact that it affects border areas. Although there is an effort to cooperate in the region, countries have different strategies and different constraints, and different results. Facing the same problem as French Guiana, neighbouring Suriname has implemented an integrated comprehensive programme funded by the global fund that has been followed by remarkable success, with the objective of malaria elimination now in sight. However, the malaria problem originates largely outside of the Surinamese territory, with most of the malaria cases reported in the Surinamese clinics in fact coming from French Guiana, thus potentially threatening the sustainability of malaria control and derailing the objective of elimination. With the “looking for gold, finding malaria” programme, Suriname has used a community-based approach with 31 malaria service deliverers working on the gold mining sites and trained in prevention, the use of rapid diagnostic tests, and the prompt delivery of quality controlled artemisinin combinations for malaria patients; this programme has been successful [8]. In addition, active case detection is performed which allows the interruption of transmission. France, however, cannot reach these invisible populations living in areas beyond the rule of law. Although malaria cases often seek treatment in Suriname or Brazil, some patients are treated by artemisinin combinations delivered by the health centres attached to Cayenne General Hospital. Patients can even benefit from costly helicopter evacuations (the first cause of emergency calls and evacuations is fever) and receive optimal treatment by infectious disease specialists. However, the core of malaria transmission and of systematic self-medication is currently not tackled by the health system. While Suriname showed flexibility, with a special focus on mobile populations, by relying on malaria service deliverers, France is constrained by national and European laws that are quite unfitting for the realities of this particular problem. In France, it is illegal for someone who is not a certified health professional to perform a rapid diagnostic test (in 2007, a decree authorized nurses or personnel from health centers to use the tests) or to prescribe a treatment. There have been various successful interventions relying on community-based approaches with trained non-professionals delivering malaria treatment [9-11], but this is not yet feasible, because not authorized in French Guiana. For health structures, sending health professionals to illegal gold mining sites is unlikely to ever take place: security there is not assured and it is probable that *garimpeiros* would believe public health workers to be no different than the government that sends forces to dismantle gold mining sites, which could put them at risk.

Thus, the fight against malaria is bound to fail if it just continues to follow the current national laws and regulations.

### Beyond the rule of law, research and action are unauthorized

The recent emergence of artemisinin resistance in *P. falciparum* in western Cambodia was detected by showing a significant reduction in parasite clearance rates and increased failure rates following artemisinin combination treatment. In addition to treatment failures and pharmacokinetic data, WHO currently recommends measuring parasite density at day 0, day 2 and day 3. The proportion of patients with a persistent parasitaemia at day 3 is a good indicator to exclude the presence of resistance. However, it is not adapted to define resistance because it depends on the initial parasite density, the timing of the sample and it requires large sample sizes. One of the pillars of the global plan to contain artemisinin resistance is to intensify monitoring and surveillance [12]. There are no reliable genetic markers for artemisinin resistance yet. For a precise assessment of resistance, a minimum of six to eight hourly monitoring of malaria parasite clearance rate, plus a follow up for a minimum of 28 days is required [13]. To do the same in France, as of today, it would be very expensive to occupy expensive hospital beds to keep patients hospitalized and legally complicated, if not illegal. The public health law forbids the conduct of interventional research on patients that do not have health insurance because, theoretically, fear of not getting treatment could coerce patients to participate in research programmes. A new law, the Jardé law, relaxes that barrier. However, the application decrees are not published yet and, until then, the Institutional Review Board’s in France still follow the Huriet Serusclat law that forbids biomedical research in persons without health insurance. For clinicians, who are in insufficient numbers in French Guiana, it seems unrealistic to expect them to decrypt the multilayered opaque French regulations that thus stand in the way of a precise assessment of resistance.

### Why is it so difficult to do what needs to be done?

The voice of French Guiana does not weigh much when European and French laws are voted. From Paris, it is hard to imagine that while at the European space port the business is booming for Arianespace, Europe is also preparing the malaria parasites of tomorrow.

For malaria experts coming from areas where most malaria detection and treatment is performed by non-doctors, the response to the malaria problem in French Guiana, a rich country, often seems puzzling: why is there no active case detection? Why does France make it so difficult to treat with primaquine (although the High Council for Public Health advising the Ministry of Health recommended it, it could only wish that a company would volunteer to initiate the process for official approval… meanwhile, some paper work and, in practice, 48 hours are necessary to obtain it), or to deal with the problem of malaria in gold miners? There is some schizophrenia in the state agencies with law enforcement clashing with public health. It is not that nothing is being done. Insecticide-treated bed nets have been funded and distributed; vector control teams are present in the villages; infectious disease specialists care for the patients seen at the hospital, Institut Pasteur does *in vitro* surveillance of drug resistance, the incidence of malaria has been divided by three in the villages of French Guiana, the regional health agency has actively pursued a regional approach with neighbouring countries. But much of the problem originates from the illegal mining sites, and the legal framework of France is not adapted for such a situation. Although the health regional agencies have benefitted from decentralized powers, they do not have the power to bypass the law, even when it is obviously not adapted to the context.

Presently, about 30,000 legal residents live in the endemic areas of French Guiana, but there are up to 15,000 illegal miners. Thus, although WHO recommends 100% coverage of exposed populations by appropriate interventions, in French Guiana it is more like two thirds of the population benefitting from a malaria programme with appropriate interventions. The rest are attempting to self medicate the problem away. Cross border efforts are not proportional. On one side of the border Suriname is following the WHO policy, on the other side France is not. Therefore, if the status quo is maintained it would be illusory to think that malaria will ever be eliminated from French Guiana, thus from the neighbouring countries of Brazil and Suriname. In the past, despite greater malaria incidence, little has been done to tackle this problem. The recent decline of malaria incidence in the villages of French Guiana will definitely not be a stimulant to make some changes: everything seems to be going well, while an invisible third of the problem is out of control.

### What could be done? Special derogations, special funds and special implementing structures for a special problem

What needs to be done is known but it cannot be done in France, under national laws.

### Reaching populations

If the health system cannot go to the gold mines, and since the gold miners would probably not spontaneously go to the military health professionals, there needs to be some way to improve detection and treatment of malaria on site. NGOs could be a solution to fill that gap. Given the number of mining sites, and their scattered locations, to cover all the ground simultaneously would be difficult and costly. Médecins du Monde, which already operates in French Guiana delivering health care for urban migrants, or Médecins sans Frontières, could be better suited to approach this problem than the official health system and its constraints. But these French NGOs may not be willing to intervene, and probably even less so in the absence of specific funds. If “professional” NGOs do not intervene, exceptional measures should allow local health authorities to implement community-based actions even if they go against some of the laws voted far away from the forests of French Guiana. Exceptional measures now, not in five years.

WHO recommends universal coverage with appropriate interventions for the entire population at risk, for prevention, diagnosis and treatment. It also emphasizes a special effort to ensure that the most vulnerable populations are covered. WHO also recommends strong leadership at the ministerial level (funding, regulations). For French Guiana, the Ministry is in Paris, malaria may not be a pressing problem there, but there should be a calibrated response to the financial and regulatory barriers that prevent the fight against malaria to reach universal coverage.

### Implementing structure

Perhaps the dispersion of health and research professionals in different institutions with different scientific pursuits and a variety of other problems to deal with is detrimental to achieve a clear focus and timely response towards the goal of controlling malaria in gold mining areas. The interventions to reach these populations should be a cross border effort since *garimpeiros* are highly mobile. The absence of a programme manager also complicates the international coordination with other national malaria programmes dealing with the same problem. The creation of an implementing structure with a voice, staff, and sufficient funds to achieve strategic goals with regards to malaria could hence facilitate this task. However, if the legal obstacles for the health system to reach gold miners remain, the added value would be marginal, because a third of the malaria problem, the most difficult third, would still not be properly dealt with.

There has been enduring concern about the emergence of unknown new pathogens from the Amazonian biodiversity hotspot [14]. One that is likely to emerge is artemisinin-resistant *P. falciparum*. Something should be done about it. Something should have been done about it much earlier because neither malaria, gold mining nor resistance to anti-malarials are particularly new [15]. In November 2012, it was reported that in Suriname, near the border with French Guiana, up to 31% of patients still had detectable parasites at day 3 after Coartem® treatment [16,17]. This needs to be confirmed, but the fear may have become reality and further delays would be disastrous.

## Competing interests

The authors declare that they have no competing interests.

## Authors’ contributions

All authors had discussions on the topic with MN. MN wrote the first draft of manuscript, PG, MD, FD, FN, BC reviewed, corrected and approved the final manuscript. All authors read and approved the final manuscript.
